# Treatment-induced neuropathy of diabetes complicated by orthostatic hypotension

**DOI:** 10.1530/EDM-25-0044

**Published:** 2025-09-11

**Authors:** Nathan Schueller, Christina Ward, Alyson Burchell, Saifuddin Nasir, Ashish Sharma

**Affiliations:** ^1^ Philadelphia College of Osteopathic Medicine, Philadelphia, USA; ^2^ UPMC Mercy Hospital, Pittsburgh, USA

**Keywords:** treatment-induced neuropathy of diabetes, glycemic control, orthostatic hypotension, neuropathy

## Abstract

**Summary:**

Treatment-induced neuropathy of diabetes (TIND) refers to the acute onset of neuropathic symptoms in patients with poorly controlled diabetes, typically as a consequence of an abrupt change in glucose levels during medical management. We present the case of a 50-year-old female with a long-standing history of poorly controlled type 2 diabetes mellitus (T2DM) complicated by progressive lower extremity weakness and associated paresthesias. Comprehensive workup was unremarkable, and symptoms could not be controlled with muscle relaxant therapies. Further chart review revealed an abrupt drop in HbA1c levels roughly 6 months before her admission. The temporal association between the rapid lowering of HbA1c and the emergence of neurological signs strongly suggested the diagnosis of TIND, which was confirmed by EMG studies. A syncopal episode further complicated the patient’s clinical course, and she was found to be orthostatic during hospital admission. The patient was started on duloxetine and given an increased dose of gabapentin, which improved her symptoms.

**Learning points:**

- TIND is an acute small-fiber neuropathy caused solely by iatrogenic factors and is often unrecognized as a complication of diabetes management.
- Neuropathic and autonomic symptoms worsen as both the magnitude and rate of change in HbA1c levels increase.
- Clinicians should be mindful of complications from rapidly lowering glucose levels and aim for an HbA1c reduction of less than 2 percentage points over 3 months.

## Background

Treatment-induced neuropathy of diabetes (TIND) is a rare, iatrogenic small-fiber neuropathy caused by the rapid lowering of glucose levels in patients with a history of poorly controlled diabetes (1, 2). Early case reports have described patients experiencing numbness, tingling, and shooting pains soon after the initiation of aggressive insulin therapy (3). TIND is defined as the acute onset of neuropathic pain and/or autonomic dysfunction within 8 weeks of lowering glycosylated hemoglobin A1C (HbA1c) levels by ≥2% points over a 3-month period (1, 2).

The true prevalence of this condition remains unclear as TIND is an underrecognized complication of diabetes (2, 4). In a single retrospective analysis, Gibbons and Freeman found that 10.9% of patients referred to a tertiary care diabetic neuropathy clinic over 5 years met criteria for the diagnosis of TIND. Many cases are not diagnosed until late in disease progression, which complicates subsequent manifestations (1, 2).

In contrast to the classical sensory–motor polyneuropathy of diabetes, TIND is characterized by a more acute onset of neuropathic pain that is usually refractory to opioids (5). In addition, TIND is frequently associated with autonomic symptoms and microvascular complications that appear concurrently or shortly after the onset of neuropathic pain. Patients commonly present with orthostatic hypotension and syncope, along with microvascular complications such as nephropathy and retinopathy (5, 6).

An important positive correlation exists between the magnitude and rate of change in HbA1c levels and the severity of symptoms. The more significant and rapid the decrease in HbA1c levels, the greater the intensity and distribution of neuropathic pain. The degree of autonomic dysfunction also correlates with the magnitude of change in HbA1c levels. This occurs independently of the agent used to correct the high HbA1c (1).

The exact pathophysiology of this acute neuropathy remains largely unknown, although two prevailing theories exist. One theory is that during periods of relative hyperglycemia, blood vessels proliferate to form arteriovenous shunts (7). As glucose levels start to decline, these new blood vessels are unable to remodel at an adequate rate, which shunts blood away from the endoneurium and causes acute neuropathy (5, 7). The fact that retinopathy and nephropathy commonly overlap with neuropathic symptoms lends credence to this theory.

An alternative theory suggests that TIND may be associated with an immune-mediated response involving proinflammatory cytokines. Rapid correction of glycemic levels in TIND could induce a transient period of relative hypoglycemia, triggering a stress response and subsequently upregulating proinflammatory cytokines. These cytokines, in turn, may damage myelinated nerves, contributing to the development of neuropathic pain, which is the hallmark of TIND.

At this stage in research, the management of TIND remains primarily supportive and emphasizes proper glycemic control. Similar to other complications of diabetes, TIND presents with a relatively poor prognosis. Both microvascular and macrovascular complications of diabetes often progress to a state of irreversibility, highlighting the importance of critical, early intervention (8). Despite the limited breadth of knowledge regarding TIND pathogenesis, it is assumed that the chronic nature of neuropathy and autonomic complications likely remain stable or worsen in patients with poor glycemic control.

## Case presentation

A 50-year-old female with a significant history of poorly controlled type 2 diabetes mellitus (DM2) complicated by peripheral neuropathy, retinopathy, hyperlipidemia, depression, anxiety, and chronic iron-deficiency anemia presented to the emergency department (ED) as a Level 1 trauma following a syncopal fall. The patient reported a week-long history of light-headedness, nausea, and progressive instability. She experienced loss of consciousness during the fall but was uncertain if she struck her head.

During syncopal evaluation, the patient denied a prior history of stroke or seizures but reported long-standing orthostatic hypotension. On admission, orthostatic testing revealed a significant blood pressure drop from 168/83 to 95/68 mmHg when moving to a sitting position. Besides blood pressure, heart rate from lying to sitting and standing positions was 81, 81, and 82, respectively. No other tests based on Ewing criteria were completed. A CT pan-scan (head, cervical, thoracic, and lumbar spine, chest, abdomen, and pelvis) was performed to rule out occult injuries and revealed no acute processes.

The patient’s acute presentation was further complicated by progressive bilateral lower extremity weakness. Her symptoms began 5 months ago with left lower extremity weakness involving the hips and ascending numbness from the calves downward. During inpatient rehabilitation (IPR) a month prior, the patient noted initial improvement in strength and sensation but later developed right-sided weakness with similar ascending progression. Over the 1.5 weeks preceding admission, the patient’s right-sided weakness deteriorated rapidly, resulting in the need for a walker. She also reported new-onset urinary incontinence.

Physical examination showed full strength in bilateral upper extremities but 1/5 strength in bilateral lower extremities. Sensation was intact to pain and light touch. Reflexes were also intact. Babinski’s sign was negative. There was no tenderness to palpation of the spine.

During the current admission, laboratory evaluations were unremarkable. Renal function: Cr 0.8, BUN 23, Hb 12.7, and Hct 34.9.

Per chart review, the patient presented 2 months earlier with bilateral lower extremity weakness and paresthesias. At that time, an MRI of the brain showed no acute infarct. Cervical and thoracic spine MRI showed minor disc osteophytes with left foraminal stenosis at C6–C7 with no significant canal stenosis in the thoracic spine. She eventually underwent an EMG study, which showed length-dependent large fiber sensory-motor mixed axonal and demyelinating peripheral neuropathy with evidence of axonal degeneration in the distal muscles. A formal diagnosis of type II diabetes mellitus was made 5 months prior, and the patient was transitioned from metformin to a combination of 15U glargine and 4U lispro TID. This adjustment caused an abrupt drop in HbA1c levels from 15.0 to 7.1. The patient was ultimately assigned a diagnosis of treatment-induced neuropathy of diabetes.

## Investigation

Emergent causes of syncopal episodes were ruled out based on unremarkable laboratory studies and imaging. The patient’s presentation of progressive lower extremity weakness without an inciting event prompted concern for Guillain-Barre syndrome (GBS) or other demyelinating conditions such as multiple sclerosis (MS) but these were subsequently ruled out with a negative workup.

## Treatment

Orthostatic hypotension improved transiently after a bladder catheterization, which relieved over 600 cc of urine. Ophthalmology consultation revealed progression of diabetic retinopathy, prompting repeat intravitreal injections. Gabapentin dose was increased, and duloxetine was initiated for neuropathic pain. The insulin regimen was optimized with increased glargine and prandial lispro to maintain glycemic control. The patient was referred for further rehabilitation and discharged to a skilled nursing facility (SNF) in stable condition. At discharge, the patient required a walker for mobility and continued to experience significant bilateral lower extremity weakness. Recommendations included follow-up with neuromuscular, ophthalmology, and primary care clinics, and continued physical therapy.

## Outcome and follow-up

The patient was admitted for a syncopal fall and was discovered to have an underlying diagnosis of TIND in light of overcorrection of HbA1c levels 5 months before the development of neurologic symptoms. Upon the most recent follow-up, the patient was readmitted to the hospital with symptoms of a UTI. In addition, her blood glucose levels did not appear to be well controlled at the SNF upon discharge, lending to potential progression of diabetic complications.

## Discussion

This case highlights the complexity of managing treatment-induced neuropathy of diabetes, particularly when complicated by autonomic dysfunction. Rapid glycemic control was associated with significant peripheral neuropathy and orthostatic hypotension, consistent with TIND.

Although the patient showed initial improvement with rehabilitation, the rapid progression of weakness and autonomic symptoms underscores the need for close monitoring and tailored therapy. Future management must balance the need for glycemic control with strategies to prevent further neurologic decline, emphasizing gradual improvement in HbA1c (8). While no clear consensus about rates of glycemic change exists, recommendations include a target change in HbA1c of <2 percentage points over 3 months (5).

Some areas of research have opposed this theory of gradual glycemic control and suggest the potential benefit of permissive hyperglycemia. This approach is hypothesized to benefit patients by intentionally reestablishing poor glycemic control, mitigating the inflammatory response associated with excessive glycemic fluctuations (9). In the context of TIND, this would likely involve deliberate glucose elevation, possibly reducing the occurrence of painful neuropathy by preventing abrupt fluctuations in glucose levels (1, 8).

However, any potential benefit must be weighed against the risks, as chronic hyperglycemia is a risk factor for end-organ damage. Further evidence in support of permissive hyperglycemia would be necessary before any change in therapeutic recommendations could be made. Until more data are available, the mainstay of treatment for TIND remains focused on symptomatic management and glucose stabilization to prevent further neuropathic deterioration (1, 8).

This case study underscores the significant gap in the literature regarding TIND and its potential progression to multisystem involvement. We emphasize the importance of clinical recognition to prevent or mitigate the progression of this acute neuropathy. By highlighting this case, we aim to draw greater attention to TIND in both clinical medicine and future research developments. Continued studies are essential to improve outcomes and develop effective disease-modifying strategies for TIND patients.

## Declaration of interest

The authors declare that there is no conflict of interest that could be perceived as prejudicing the impartiality of the work reported.

## Funding

This research did not receive any specific grant from any funding agency in the public, commercial, or not-for-profit sector.

## Patient consent

Written informed consent for publication of their clinical details and/or clinical images was obtained from the patient/parent/guardian/relative of the patient.
